# Yeast Cell Wall Extract Induces Disease Resistance against Bacterial and Fungal Pathogens in *Arabidopsis thaliana* and *Brassica* Crop

**DOI:** 10.1371/journal.pone.0115864

**Published:** 2015-01-07

**Authors:** Mari Narusaka, Taichi Minami, Chikako Iwabuchi, Takashi Hamasaki, Satoko Takasaki, Kimito Kawamura, Yoshihiro Narusaka

**Affiliations:** 1 Research Institute for Biological Sciences Okayama, Okayama, Japan; 2 Research & Development Laboratories for Sustainable Value Creation, Asahi Group Holdings, Ltd., Ibaraki, Japan; UMBC, UNITED STATES

## Abstract

Housaku Monogatari (HM) is a plant activator prepared from a yeast cell wall extract. We examined the efficacy of HM application and observed that HM treatment increased the resistance of *Arabidopsis thaliana* and *Brassica rapa* leaves to bacterial and fungal infections. HM reduced the severity of bacterial leaf spot and anthracnose on *A. thaliana* and *Brassica* crop leaves with protective effects. In addition, gene expression analysis of *A. thaliana* plants after treatment with HM indicated increased expression of several plant defense-related genes. HM treatment appears to induce early activation of jasmonate/ethylene and late activation of salicylic acid (SA) pathways. Analysis using signaling mutants revealed that HM required SA accumulation and SA signaling to facilitate resistance to the bacterial pathogen *Pseudomonas syringae* pv. *maculicola* and the fungal pathogen *Colletotrichum higginsianum*. In addition, HM-induced resistance conferred chitin-independent disease resistance to bacterial pathogens in *A. thaliana*. These results suggest that HM contains multiple microbe-associated molecular patterns that activate defense responses in plants. These findings suggest that the application of HM is a useful tool that may facilitate new disease control methods.

## Introduction

In nature, numerous potential pathogens such as fungi, bacteria, and viruses continually attack plants; however, disease development remains the exception. Plants are immune to most potential pathogens; this characteristic is referred to as a “nonhost resistance.” Moreover, they have the ability to reduce the disease severity of actual pathogens. Plant innate immune responses to pathogens consist of a two-layer surveillance system that comprises pattern recognition receptors (PRRs) and intracellular nucleotide binding-leucine rich repeat (NLR) proteins, which are encoded by *R* (resistance) genes [1]. PRRs, which are localized on the surface of plant cells, recognize microbe- or pathogen-associated molecular patterns (MAMPs or PAMPs), and intracellular NLR-type R proteins subsequently detect effectors secreted by the pathogens inside the cell. These two phases of defense induction are called MAMP- or PAMP-triggered immunity and effector-triggered immunity [2].

MAMPs are common conserved structures in microbes among pathogenic, nonpathogenic, and saprophytic microorganisms. They include chitin and ergosterol, which are the main structural components of cell walls and membranes in higher fungi, respectively; bacterial lipopolysaccarides, which are glycolipid components of the outer membranes of gram-negative bacteria; and flagellin, which is the major structural component of the bacterial motility organ [3].

MAMP-induced early defense responses include ion fluxes across the plasma membrane (Ca^2+^ and H^+^ influx) and the generation of reactive oxygen species, reactive nitrogen species, and ethylene (ET). Later MAMP responses include alterations in the plant cell wall (deposition of callose and oxidative cross-linking of polymers), biosynthesis of antimicrobial compounds (alkaloids, flavonoids/isoflavonoids, phytoalexin, terpenes, and others), and expression of *pathogenesis-related* (*PR*) genes. In addition, MAMPs trigger the activation of calcium-dependent protein kinases and mitogen-activated protein kinase cascades, which lead to transcriptional changes in numerous genes [4–6].

The budding yeast, *Saccharomyces pastorianus*, is the bottom fermentation yeast used in brewing. Yeast extract and a mannopeptide from yeast invertase function as MAMP and elicit plant defense responses [7, 8]. However, the yeast cell wall extract (YCWE), a by-product of beer brewing processes, has not been effectively used as an agricultural chemical. The yeast cell wall mainly consists of polysaccharides such as polymers of glucose (β-glucan) and polymers of mannose (mannoproteins) [9], which may act as MAMPs and subsequently induce plant defense responses. Using YCWE, which has been processed by treatment with cell wall-degrading enzymes, as a main ingredient, Housaku Monogatari (HM) has been developed as a compound fertilizer (http://www.asahi-fh.com/products/wholesale/good-harvest/).

Our previous study indicated that HM led to induced resistance, i.e., systemic acquired resistance (SAR) and induced systemic resistance signaling pathways [10]. However, the mechanism that enables HM to activate systemic resistance remains unknown. In the present study, we investigated the induction of the plant immune system and changes in the expression of pathogen-responsive genes following HM treatment. We observed that HM activates salicylic acid (SA) and jasmonate (JA)/ET defense signaling pathways and that HM is effective against bacterial pathogens.

## Results

### HM-induced gene expression

Induced resistance is associated with the expression of disease resistance marker genes. To determine whether HM acts as an inducer of induced resistance in *Arabidopsis thaliana*, we investigated the expression profile of defense-related genes in response to HM treatment in *A. thaliana* Col-0 plants by quantitative real time-polymerase chain reaction (qRT-PCR). We used the *A. thaliana PR-1* gene as a marker for the SA-dependent signal transduction pathway [11] and the *PDF1.2* gene, which encodes a plant defensin, as a marker for the JA/ET pathway [12].

**Figure 1:**
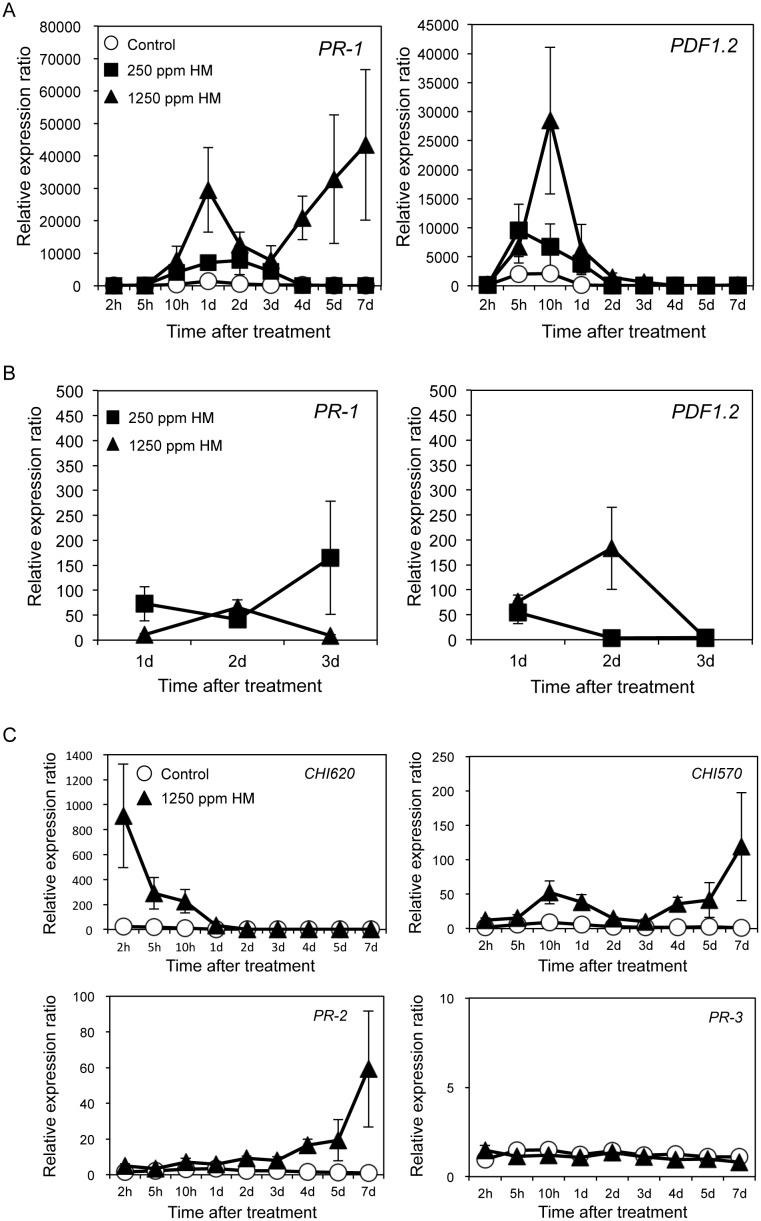
Expression of defense-related genes after treatment with HM. The 28- to 30-day-old *Arabidopsis thaliana* Col-0 plants were sprayed (A, C) or soil drenched (B) with 250 and/or 1250 ppm. Aboveground tissues were collected 0–7 (A, C) or 0–3 (B) days after treatment. Total RNA was extracted, and first-strand cDNA was synthesized for expression analysis. Expression levels of the *PR-1*, *PDF1.2, PR-2* (*glucanase*; *BGL2*), *basic chitinase PR-3*, and *chitinase* (At2g43620; *CHI620*, At2g43570; *CHI570*) genes were monitored by qRT-PCR. The expression level of each gene was normalized against the expression level of *CBP20*, which is constitutively expressed. Relative expression ratios are shown as fold induction relative to the expression level at 0 h. Each experiment was repeated at least three times. Bars indicate the standard error (SE). The nucleotide sequences of the gene-specific primers for each gene are listed in Table 1.

Expression of the *PR-1* gene increased over time in Col-0 plants sprayed with 1250 ppm HM, with a peak at day 1, whereas the expression level continued to rise for 2 days in plants sprayed with 250 ppm HM (Fig. 1A). The expression of *PDF1.2* initially increased with a peak at 10 h and 5 h in Col-0 plants sprayed with 1250 ppm and 250 ppm HM, respectively, which was earlier than that of the *PR-1* gene. The timing of induction differed between the two representative marker genes.

**Table 1:** PCR primers used in the present study.

	**Forward primer**	**Reverse primer**
***PR-1***	CCC ACA AGA TTA TCT AAG GGT TCA C	CCC TCT CGT CCC ACT GCA T
***PDF1.2***	CCA TCA TCA CCC TTA TCT TCG C	TGT CCC ACT TGG CTT CTC G
***PR-2***	CTT CAA CCA CAC AGC TGG A	GCA TTC GCT GGA TGT TTT GT
***PR-3***	GGC AAA CGC TAC TAC GGA AG	AAG CGA TCA CTG CGT CGT T
***CHI620***	GCT AGA GGG AAA TAC TGC TCA C	GAG TCC GAG GAA CTT TCC AG
***CHI570***	CCA AGA AAC AGG GTT CAT GTG T	TAG TAG CCC TTT CCT TGT GC

The expression of the *PR-1* gene increased with time in Col-0 plants where the soil was drenched with 250 ppm HM (Fig. 1B). In addition, the expression of *PR-1* and *PDF1.2* increased with a peak at 2 days in Col-0 plants where the soil was drenched with 1250 ppm HM. The transcription levels of *PR-1* and *PDF1.2* were much higher in the Col-0 plants sprayed with HM than in plants where the soil was drenched.

To investigate the signal transduction pathways activated by HM, we examined other defense-related genes induced in response to treatment with HM using *A. thaliana* as a model system. Thus, we used qRT-PCR to analyze the effects of HM on the expression of the following *A. thaliana* putative marker genes: *PR-2* (*glucanase*; *BGL2*), *basic chitinase PR-3* (At3g12500), and *chitinase* (At2g43620; *CHI620*, At2g43570; *CHI570*). The expression levels of these genes were induced by 1250 ppm HM treatment (Fig. 1C). The expression levels of *chitinase* (*CHI620*, *CHI570*) and *glucanase* (*PR-2*), which are antipathogenic enzymes, also increased after HM treatment. The expression level of *CHI620* increased early in Col-0 plants sprayed with HM. Thus, the plants recognized the HM components as MAMPs and increased the expression of defense-related genes.

### HM protects *Arabidopsis* against *Pseudomonas syringae* pv. *maculicola*

**Figure 2:**
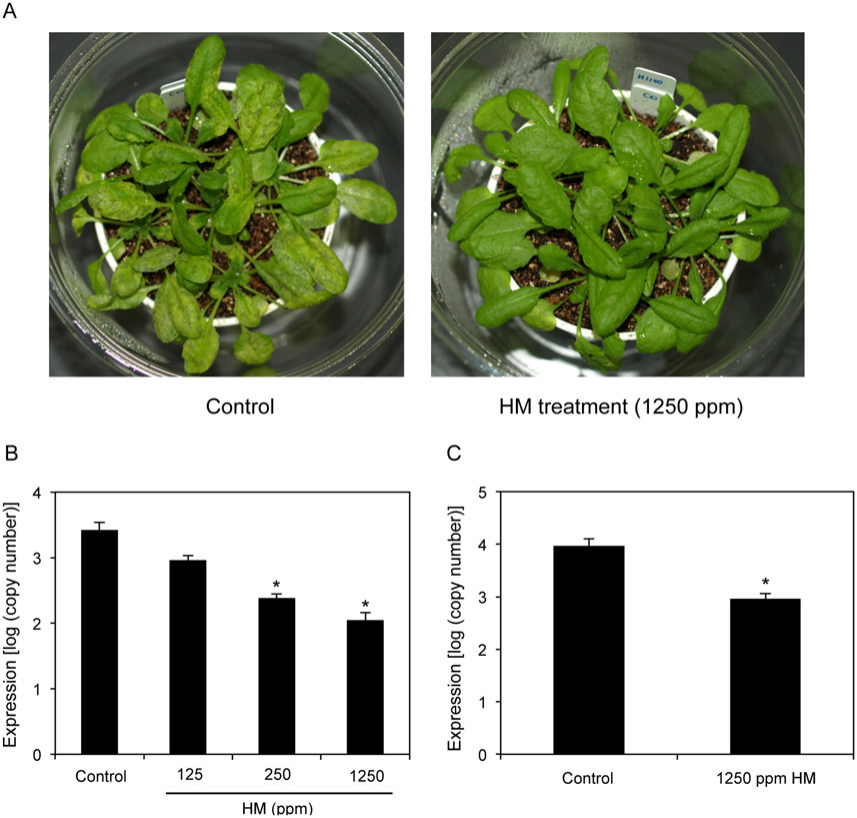
Effects of HM application on *P. syringae* pv. *maculicola* in *Arabidopsis* plants. The 35-day-old *A. thaliana* Col-0 plants were sprayed (A, B) or soil drenched (C) with water (control) or the indicated concentrations of HM at 2 days prior to spray inoculation with a bacterial suspension (10^8^ cfu mL^−1^) of *P. syringae* pv. *maculicola*. Symptoms were observed 3 days after inoculation with the pathogen (A). Pathogen growth was determined 3 days after inoculation by assessing *P. syringae* pv. *maculicola-rpoD* mRNA by qRT-PCR (B, C). Bars indicate the standard error (SE). The asterisk indicates a significant difference compared with the control (Dunnett’s method [35], *P* < 0.05). The experiment was repeated at least twice with similar results.

Induced resistance is characterized by the induction of disease resistance to various pathogens. Previously, induced resistance in *A. thaliana* has been shown to be effective against disease caused by *P. syringae* pv. *tomato* [13]. To determine whether HM acts as an inducer of induced resistance in *A. thaliana* Col-0, the plants were sprayed with water or HM 2 days prior to *P. syringae* pv. *maculicola* (*Psm*) inoculation. As shown in Fig. 2A, treatment with 1250 ppm HM decreased the disease symptoms caused by the pathogen. In contrast, water-treated plants inoculated with *Psm* exhibited chlorotic leaf spotting. In addition, bacterial growth was reduced in Col-0 plants sprayed with 1250 ppm HM. At 3 days postinoculation (dpi), the HM-treated plants contained 10-fold lower bacterial titers than the water-presprayed control leaves (Fig. 2B). Thus, reduced bacterial growth was responsible for the decrease in the disease symptoms. Furthermore, this concentration of HM did not cause any phytotoxicity. Moreover, bacterial growth decreased in Col-0 plants where the soil was drenched with 1250 ppm HM (Fig. 2C).

To determine whether HM treatment reduced bacterial growth in a dose-dependent manner, HM was applied at increasing concentrations and bacterial growth was measured (Fig. 2B). Compared with the control, HM concentrations as low as 250 ppm reduced bacterial growth in a dose-dependent manner.

**Figure 3:**
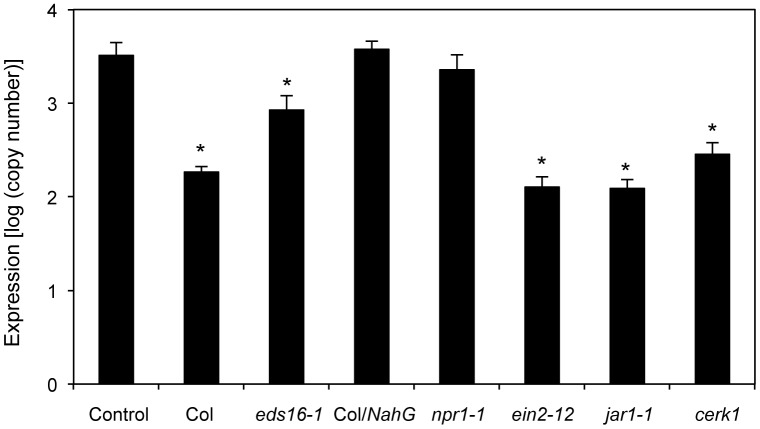
Induction of resistance to *P. syringae* pv. *maculicola* by HM application in *Arabidopsis* defense signaling-defective mutants. The 35-day-old *A. thaliana* mutants were sprayed with water (control) or 1250 ppm HM at 2 days prior to spray inoculation with a bacterial suspension (10^8^ cfu mL^−1^) of *P. syringae* pv. *maculicola*. Pathogen growth was determined 3 days after inoculation by assessing *P. syringae* pv. *maculicola-rpoD* mRNA by qRT-PCR. Bars indicate the standard error (SE). The asterisk indicates a significant difference compared with the control (Dunnett’s method [35], *P* < 0.05). The experiment was repeated at least three times with similar results.

We investigated whether the defense-related hormone SA plays a role in HM-induced activation of SAR using *NahG* transgenic *Arabidopsis*, which fails to accumulate SA [14]; *eds16–1*, which does not produce SA [15]; and *npr1–1*, which fails to activate *PR* gene expression [16]. Both JA and ET have been suggested to play important roles in plant defense against pathogen infection. To determine the roles of JA and ET in HM-induced resistance, we tested *A. thaliana* mutants that varied in their ability to respond to methyl jasmonate (*jar1–1* is insensitive to JA [17]) or ET (*ein2–12* is insensitive to ET [18]). HM was applied to these plants, and their resistance levels to *Psm* were determined. These mutants were treated with 1250 ppm HM at 2 days prior to bacterial inoculation. At 3 dpi, mutants deficient in the SA signaling pathways exhibited decreased induced resistance to *Psm* infection, whereas mutants deficient in the JA or ET signaling pathways were protected in a manner similar to the wild-type Col-0 (Fig. 3). The water-pretreated controls exhibited increased bacterial growth. Thus, HM appears to activate disease resistance to *Psm* via a pathway that is dependent on SA. In addition, HM has no direct toxic effect on bacteria.

**Figure 4:**
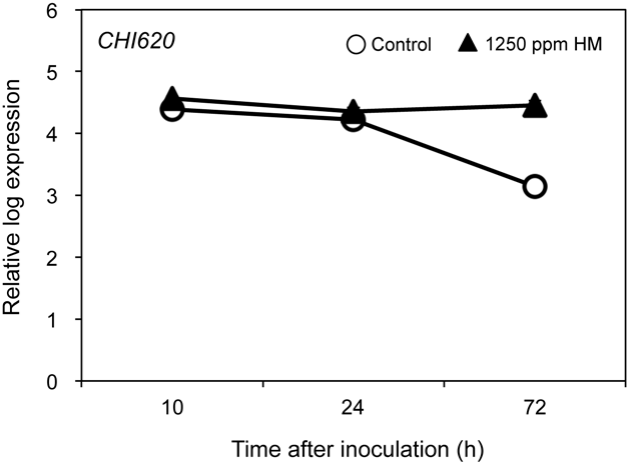
Expression of *chitinase* genes in HM-treated *Arabidopsis* plants during infection with *P. syringae* pv. *Maculicola*. The 35-day-old *A. thaliana* Col-0 plants were sprayed with water (control) or 1250 ppm HM at 2 days prior to spray inoculation with a bacterial suspension (10^8^ cfu mL^−1^) of *P. syringae* pv. *maculicola*. Aboveground tissues were collected 10, 24, 72 h after inoculation. Total RNA was extracted, and first-strand cDNA was synthesized for expression analysis. Expression levels of the *chitinase* gene (At2g43620; *CHI620*) were monitored by qRT-PCR. The expression level of each gene was normalized against the expression level of *CBP20*, which is constitutively expressed. Each experiment was repeated at least twice with similar results. Bars indicate the standard error (SE). The nucleotide sequences of the gene-specific primers for each gene are listed in Table 1.

We investigated whether HM potentiates defense responses during infection with pathogens. Treatment with HM potentiated *Arabidopsis* plants inoculated with *Psm* to induce expression of *chitinase* (*CHI620*) gene more intensively (Fig. 4). Thus, defense responses are boosted in HM-treated plants during the infection with *Psm*.

Furthermore, the expression of *PR-1* and *PDF1.2* genes in SA/ET/JA signaling-defective mutants were investigated for analyzing HM-primed defense responses after infection with *Psm*.

**Figure 5:**
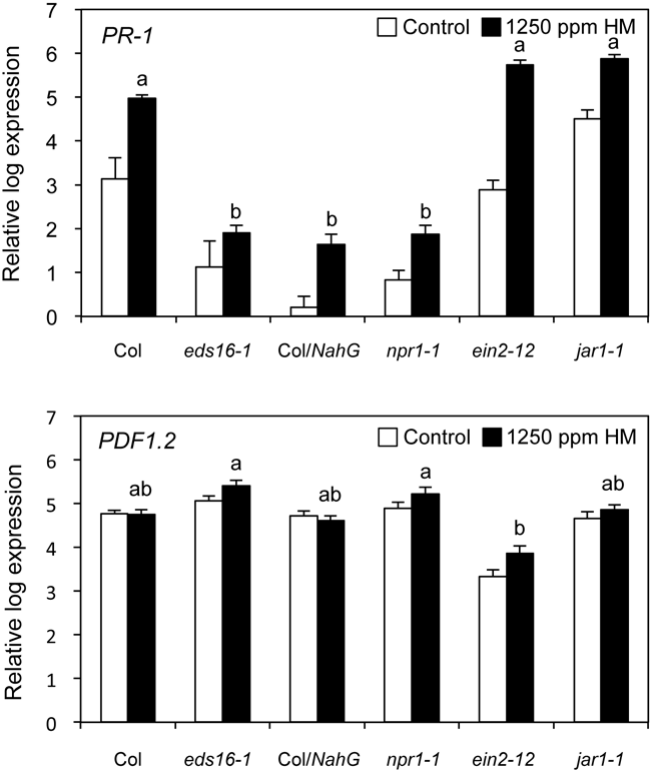
HM-primed defense responses after the infection with *P. syringae* pv. *maculicola* in *Arabidopsis* defense signaling-defective mutants. The 35-day-old *A. thaliana* Col-0 plants and defense signaling-defective mutants were sprayed with water (control) or 1250 ppm HM at 2 days prior to spray inoculation with a bacterial suspension (10^8^ cfu mL^−1^) of *P. syringae* pv. *maculicola*. Aboveground tissues were collected 10 h after inoculation. Total RNA was extracted, and first-strand cDNA was synthesized for expression analysis. Expression levels of the *PR-1* and *PDF1.2* genes were monitored by qRT-PCR. The expression level of each gene was normalized against the expression level of *CBP20*, which is constitutively expressed and is shown as the reference value. Each experiment was repeated at least twice with similar results. Bars indicate the standard error (SE). Means labeled with the same letters are not statistically different at the 5% confidence level based on Tukey’s test. The nucleotide sequences of the gene-specific primers for each gene are listed in Table 1.

Accumulation of *PR-1* transcripts in *NahG* transgenic plants, *eds16–1* and *npr1–1* mutants were lower than those in Col-WT, *ein2–12* and *jar1–1* mutants during the early phase of infection (10 h post inoculation) (Fig. 5). In addition, *PDF1.2* transcripts accumulated in *NahG*, *eds16–1* and *npr1–1* plants, but accumulation of *PDF1.2* transcripts was low level in *ein2–12* mutant (Fig. 5). Therefore, accumulation of *PR-1* mRNAs depended on activation of SA signaling defense pathways during infection with *Psm*.

### Does HM activate chitin signaling?

Chitin is a representative MAMP molecule for various fungi, and its perception by PRRs triggers various plant defense responses [19, 20]. Chitin elicitor receptor kinase 1 (CERK1) is an essential molecule for chitin perception and chitin elicitor signaling in *A. thaliana* [21]. In the present study, compared with control plants, an HM-treated *cerk1* mutant exhibited increased resistance to *Psm* (Fig. 3).

### HM-induced disease resistance to *Colletotrichum higginsianum*

**Figure 6:**
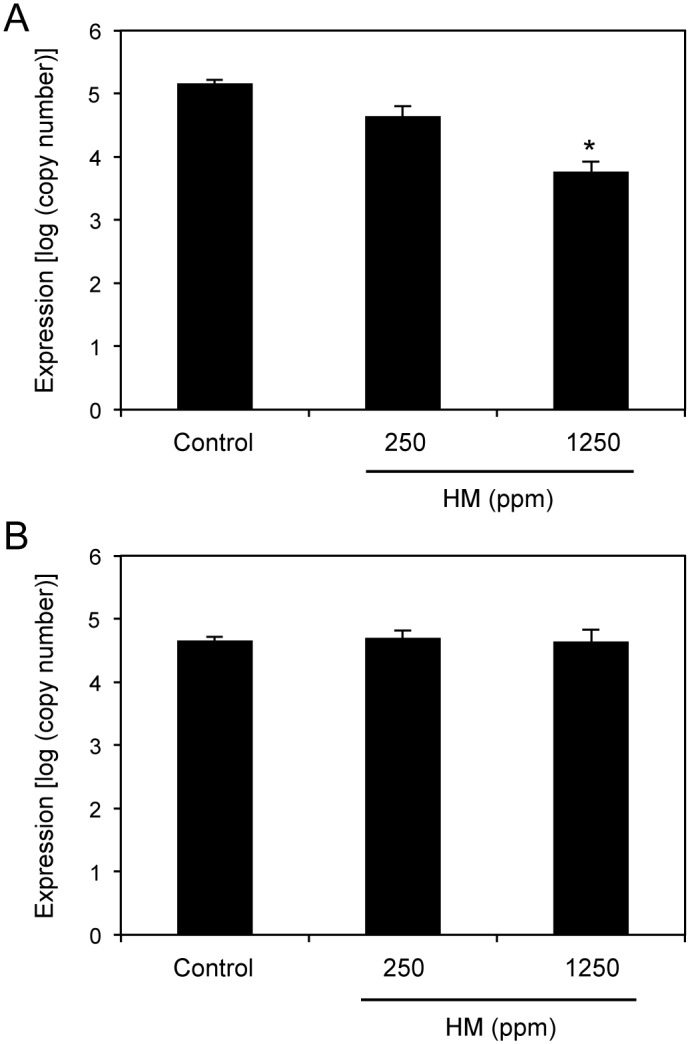
Effect of HM application on *Colletotrichum higginsianum* in *Arabidopsis* plants. The 28- to 30-day-old *A. thaliana* Col-0 plants were sprayed (A) or soil drenched (B) with water (control) or the indicated concentrations of HM at 2 days prior to spray inoculation with a spore suspension (5 × 10^5^ spores mL^−1^) of *C. higginsianum*. Pathogen growth was determined 5 days after inoculation by assessing *C. higginsianum actin* mRNA by qRT-PCR. Bars indicate the standard error (SE). The asterisk indicates a significant difference compared with the control (Dunnett’s method [35], *P* < 0.05). The experiment was repeated at least twice with similar results.

To test whether HM acts as an inducer of resistance to fungal pathogens, *A. thaliana* plants were sprayed with water or HM at 2 days prior to *C. higginsianum* inoculation. The results showed that the infection process was not completely stopped by HM treatment; however, the disease incidence was moderately reduced (Fig. 6A). In contrast, no enhanced resistance to *C. higginsianum* was detected in the Col-0 plants grown in soil drenched with 1250 ppm HM (Fig. 6B).

**Figure 7:**
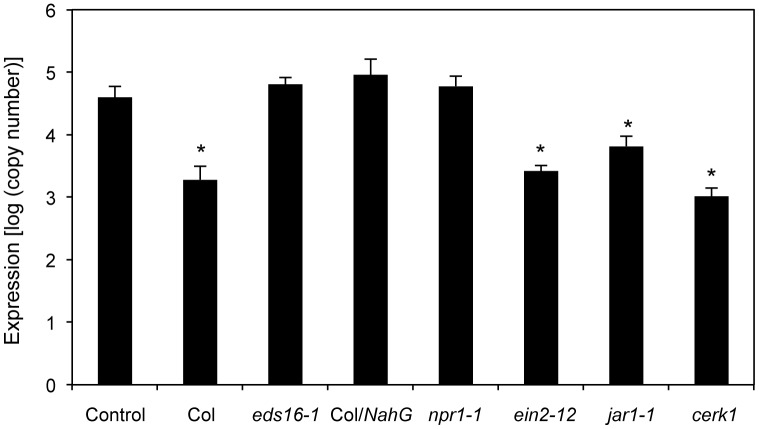
Induction of resistance to *C. higginsianum* by HM application in *Arabidopsis* defense signaling-defective mutants. The 28- to 30-day-old *A. thaliana* mutants were sprayed with water (control) or 1250 ppm HM at 2 days prior to spray inoculation with a spore suspension (5 × 10^5^ spores mL^−1^) of *C. higginsianum*. Pathogen growth was determined 5 days after inoculation by assessing *C. higginsianum actin* mRNA by qRT-PCR. Bars indicate the standard error (SE). The asterisk indicates a significant difference compared with the control (Dunnett’s method [35], *P* < 0.05). The experiment was repeated at least twice with similar results.

HM was applied to transgenic plants and mutants, and their resistance to *C. higginsianum* was evaluated (Fig. 7). Enhanced resistance to *C. higginsianum* was not detected in *NahG* transgenic plants and *eds16–1* and *npr1–1* mutants that had been pretreated with 1250 ppm HM at 2 days prior to fungal inoculation. In contrast, HM-treated *ein2–12* and *jar1–1* mutants did not support the fungal growth, whereas water-pretreated controls exhibited increased fungal growth. Thus, HM appears to activate disease resistance to *C. higginsianum* via an SA-dependent pathway. Compared with control plants, the HM-treated *cerk1* mutant also exhibited increased resistance to *C. higginsianum* (Fig. 7). In addition, HM exhibited no direct toxic effect on fungi.

### HM protects *Brassica* crops against *P. cannabina* pv. *alisalensis*

**Figure 8:**
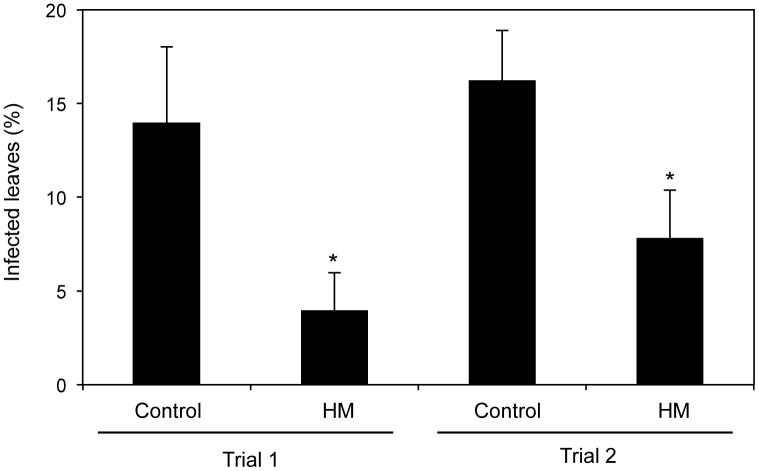
HM application induced resistance against *P. cannabina* pv. *alisalensis* in *Brassica rapa* var. *chinensis*. *Brassica rapa* var. *chinensis* seeds were sown in soil mixed with 5 g L^−1^ of raw HM material powder. Two-week-old seedlings were inoculated at approximately the one true leaf stage by spraying with a bacterial suspension (10^6^ cfu mL^−1^) of *P. cannabina* pv. *alisalensis*. Observations of bacterial leaf spot symptoms were made at 7 dpi. The percentage of infected leaves (IL) was calculated as follows: IL = (number of leaves with any chlorotic leaf spots)/(total number of leaves), Data represent mean ± SE (Trial 1: *n* = 12, Trial 2: *n* = 23). Asterisks indicate significant differences compared with the controls (Welch’s *t*-test [36]).

*P. syringae* pv. *alisalensis* was recently renamed as *P. cannabina* pv. *alisalensis* (*Pca*) [22]. To test whether HM induces resistance in *Brassica rapa* var. *chinensis*, plants treated with HM were inoculated by spraying with *Pca*. The results revealed that HM protects *Brassica* crops against *Pca* (Fig. 8). Thus, HM appears to activate disease resistance in *Brassica* crops.

## Discussion

YCWE has not been tested in practice as an agricultural chemical. However, it may act as MAMP and lead to induced resistance in plants. The mode of action of YCWE is not well known in plants; therefore, we analyzed the protective effect of this product against bacterial and fungal pathogens in *A. thaliana* and *Brassica* plants. In *A. thaliana* and *Brassica* plants, we observed that HM prepared from YCWE induced the expression of plant defense-related genes and resistance to *Psm* and *Pca*, which are similar pathogens that are combated via SA-induced resistance. The infection process of the hemibiotrophic fungal pathogen *C. higginsianum* was not completely stopped by HM treatment; however, plants treated with HM were moderately protected against the pathogen. Thus, HM protects different Brassicaceae species against bacterial and fungal pathogens, demonstrating the broad range of activity of this product. Importantly, HM has no direct toxic effect on bacteria or fungi. HM-mediated resistance is probably based on the activation of host resistance mechanisms.

Yeast cell walls comprise polysaccharides, i.e., approximately 40% of mannoproteins, approximately 60% of β-glucan, and approximately 2% of chitin [9, 23], whereas HM contains approximately 1.3% of chitin. Chitin-induced defense responses were completely suppressed in the *cerk1* mutant [21]; thus, the present study indicated that HM-induced resistance involved chitin-independent disease resistance to bacterial and fungal pathogens in *A. thaliana*.

The *NahG* transgenic plants and *eds16–1* and *npr1–1* mutants exhibited reduced induced resistance to *Psm* and *C. higginsianum* after HM treatment, indicating that SA synthesis and NPR1 play a role in HM-elicited defense responses of *A. thaliana* against *Psm* and *C. higginsianum*. In addition, the results demonstrated that HM does not exhibit a direct antibiotic effect on *Psm* and *C. higginsianum*. The impaired synthesis of SA, EDS16, and NPR1 in defense mechanisms may be disadvantageous for plants. In contrast, the defective mutants of JA and ET signaling pathways, *jar1–1* and *ein2–12*, respectively, were protected against *Psm* and *C. higginsianum* with an effect resembling the wild-type Col-0 control. The plant hormones JA and ET have been implicated in an alternative defense transduction pathway that is separate from SA [24]. HM-induced resistance to these pathogens was not dependent on the sensitivity to JA and ET, which excludes the involvement of JA- and ET-mediated defense signaling mechanisms. A previous study revealed that β-aminobutyric acid (BABA), which is known to be a disease resistance inducer, did not protect transgenic *Arabidopsis NahG* plants and *npr1–1* mutants against *P. syringae* pv. tomato DC3000 (*Pst* DC3000) [25]. Lawton et al. revealed that benzothiadiazole (BTH)-treated *A. thaliana* plants exhibited resistance to *Pst* DC3000 [26]. In addition, BTH treatment induced disease resistance in *NahG* transgenic *A. thaliana* plants, suggesting that the action of BTH does not require SA accumulation [26]. However, in contrast to BTH, HM and BABA require SA accumulation and enhance resistance via plant defense responses.

The protective action of HM against *Psm* and *C. higginsianum* seems to act as a disease resistance inducer or a plant defense activator but not as a bactericide and fungicide. Therefore, we analyzed the expression of plant defense-related genes induced by HM treatment. The results indicated that HM treatment elicited the induction of several defense-signaling pathways, including SA, JA, and ET signaling. There is antagonistic signaling cross-talk between the defense signal transduction pathways in *A. thaliana* and tobacco [27–30]. The maximum induction timing for two representative marker genes of plant defense responses, *PR-1* and *PDF1.2*, differed in *A. thaliana* treated with HM. Thus, HM treatment appeared to induce early activation of the JA/ET signaling pathway and late activation of the SA signaling pathway. The timing of activation of these signaling pathways may be essential for plant resistance to pathogenic infections. Therefore, the activation of SA and JA/ET defense signaling pathways at different times and our analyses using signaling mutants of these pathways suggest that HM could control various diseases.

HM-primed and -induced defense responses after the infection with *Psm* were investigated by analyzing the expression of defense-related genes in defense signaling-defective mutants and wild-type plants. The expression of *PR-1* gene in response to *Psm* was reduced in HM-treated SA signaling-defective mutants indicating susceptible to *Psm*. On the contrary, *PR-1* transcripts were potentiated in ET/JA signaling-defective mutants indicating resistance to *Psm*. As a consequence, SA-mediated defense responses are involved in resistance to *Psm*.

In addition, expression of *CHI620* gene was strongly induced in resistance interaction of HM-treated plants with *Psm*, but weekly induced in susceptible interaction. Therefore, HM treatment prime and boost defense responses leading to restriction of *Psm* growth.

Our results indicate that HM reduced the severity of bacterial leaf spot and anthracnose on *A. thaliana* and *Brassica* crop leaves with protective effects. In addition, HM facilitated induced resistance to pathogens on plant leaves, which enhanced the expression of plant defense-related genes, i.e., *PR* gene expression. Our experiments suggest that HM could be a useful tool in the effective control of plant disease.

## Materials and Methods

### Preparation of Housaku Monogatari (HM)

HM was produced using a by-product, the yeast cell wall extract (YCWE), prepared from the budding yeast *Saccharomyces pastorianus* during the beer brewing process. The yeast slurry collected after beer brewing was treated with protease YL-15 (Amano Enzyme Inc., Nagoya, Japan). YCWE was obtained by removing the supernatant following centrifugation. To digest YCWE to a moderate molecular size, YCWE was treated again with 0.5% YL-15 at 55°C for 18 h and powdered HM was produced by drum drying. Similar to general yeast cell wall composition [9], HM contains polysaccharides (15%–25% of β-glucan, 5%–15% of α-glucan, 10%–20% of mannan, and 0.5%–2% of chitin).

### Plant materials

*Arabidopsis thaliana* accession Columbia-0 (Col-0) was obtained from RIKEN BRC (Tsukuba, Japan). Before inoculation, the plants were grown in Soil-mix (Sakata Seed Corp., Yokohama, Japan) and expanded vermiculite (1.5–2 mm granules) at a ratio of 1:2 for 28–35 days in a growth chamber at 22°C with 12-h light.

*Brassica rapa* var. *chinensis* (cv. Seitei, Sakata Seed Co., Yokohama, Japan) plants were grown in Yosaku N-15 (JCAM AGRI.CO., LTD, Tokyo, Japan) in a growth chamber with 75% relative humidity at 26°C during the daylight hours.

### HM treatment

*A. thaliana* plants were treated with water (control) or the indicated concentration of HM, containing a spreader (0.01%) for spraying but not for soil drenching.

### Expression analysis of defense-related genes in *Arabidopsis* plants

The expression levels of the *PR-1* and *PDF1.2* genes were monitored by quantitative real time-polymerase chain reaction (qRT-PCR), as described previously [31]. *PR-2* (*glucanase*; *BGL2*), *basic chitinase PR-3*, and *chitinase* (At2g43620; *CHI620*, At2g43570; *CHI570*) gene expression levels were also monitored by qRT-PCR. The primers used for qRT-PCR are listed in Table 1.

### *Pseudomonas syringae* pv. *maculicola* infection and quantification of *rpoD* mRNA

Spontaneous rifampicin-resistant colonies of *P. syringae* pv. *maculicola* (MAFF302783Rif4) (*Psm*) were obtained by culturing the strain MAFF302783 in King’s B medium containing 100 μg mL^−1^ of rifampicin. *Psm* was grown in liquid King’s B medium containing rifampicin (25 μg mL^−1^). Bacteria were harvested by centrifugation, and the cell pellets were washed with 10 mM MgSO_4_ before resuspension in 10 mM MgSO_4_ at a concentration of 1 × 10^8^ cfu mL^−1^ for *in planta* growth assays. Five-week-old *Arabidopsis* Col-0 plants (susceptible to *Psm*) and the mutants were used in virulence assays. The plants were inoculated by spraying the leaves with the bacterial suspension. The inoculated plants were then placed in a growth chamber with 100% relative humidity at 22°C (12-h light cycle). Bacterial growth was determined 3 days after inoculation, and pathogen growth was determined by measuring the *rpoD* mRNA level by qRT-PCR, as described previously [32].

### *Colletotrichum higginsianum* infection and quantification of *actin* mRNA

*Colletotrichum higginsianum* Saccardo isolates (MAFF305635) were obtained from the MAFF Genebank project, Japan. The 28- to 30-day-old *Arabidopsis* Col-0 plants were inoculated as described previously [33].

Plants inoculated with *C. higginsianum* were harvested at 5 days postinoculation (dpi) for qRT-PCR, and *C. higginsianum* was quantified as described previously [34].

### *P. cannabina* pv. *alisalensis* infection

*B. rapa* var. *chinensis* seeds were sown in soil mixed with 5 g L^−1^ of HM raw material powder. Two-week-old seedlings at approximately the one true leaf stage were inoculated by spraying with a bacterial suspension (10^6^ cfu mL^−1^) of *P. cannabina* pv. *alisalensis*. Observations of bacterial leaf spot symptoms were made at 7 dpi. The percentage of infected leaves (IL) was calculated as follows:

IL = (number of leaves with any chlorotic leaf spots)/(number of leaves)
